# Selective Targeting of IL‐1RAP‐Dependent Eosinophilic Inflammation in Allergic Fungal Airway Disease

**DOI:** 10.1111/all.70256

**Published:** 2026-02-11

**Authors:** Thomas J. Williams, James S. Griffiths, Luis E. Gonzales‐Huerta, David Bell, Anna K. Reed, Anand Shah, Julian R. Naglik, Darius Armstrong‐James

**Affiliations:** ^1^ Department of Infectious Disease, Faculty of Medicine Imperial College London London UK; ^2^ Department of Cardiothoracic Transplantation and Mechanical Support, Harefield Hospital, Royal Brompton and Harefield Hospitals Guy's and St Thomas' NHS Foundation Trust London UK; ^3^ Centre for Host‐Microbiome Interactions (CHMI), Faculty of Dentistry, Oral and Craniofacial Sciences, Kings College London London UK; ^4^ Centre for Synthetic Biology Imperial College London London UK; ^5^ SynbiCITE Innovation and Knowledge Centre Imperial College London London UK; ^6^ Department of Respiratory Medicine, Royal Brompton and Harefield Hospitals Guy's and St Thomas' NHS Foundation Trust London UK

To the Editor,

*Aspergillus fumigatus* (Af) is a widespread airborne mould and a major cause of asthma complications like severe asthma with fungal sensitization (SAFS) and allergic bronchopulmonary aspergillosis (ABPA), impacting over 10 million people worldwide [1]. Recent murine models using repeated Af conidia exposure better emulate clinical disease than older extract‐based methods, allowing improved modelling of fungal allergy [2]. The interleukin‐1 (IL‐1) family including IL‐1β and IL‐33 drives inflammation via IL‐1RAP [3]. Recent biologics targeting IL‐1RAP have demonstrated efficacy in inflammatory models and represent promising candidates for allergic airway diseases. Our findings demonstrate the important role of IL‐1RAP signalling in driving type 2 inflammation in allergic fungal airway disease, showing that the absence of IL‐1 family signalling results in an attenuated, but not absent, allergic phenotype.

Here, using C57BL/6J mice we characterised a repeat challenge model of allergic fungal airway disease, in which animals were given repeated low doses of live conidia over a two‐week period. No mortality or significant weight loss occurred; however, marked inflammation, mucus overproduction, and cellular infiltrates were evident (Figure S1A–E), while low‐level fungal persistence in the airways was observed (Figure S1F). Analysis of airway leukocytes revealed progressive eosinophilia, comprising approximately 75% of BAL cells by day 14, accompanied by sustained neutrophil and monocyte influx, loss of resident alveolar macrophages, and significant T and B cell increases (Figure 1A–C and Figure S1G–I).

**FIGURE 1:**
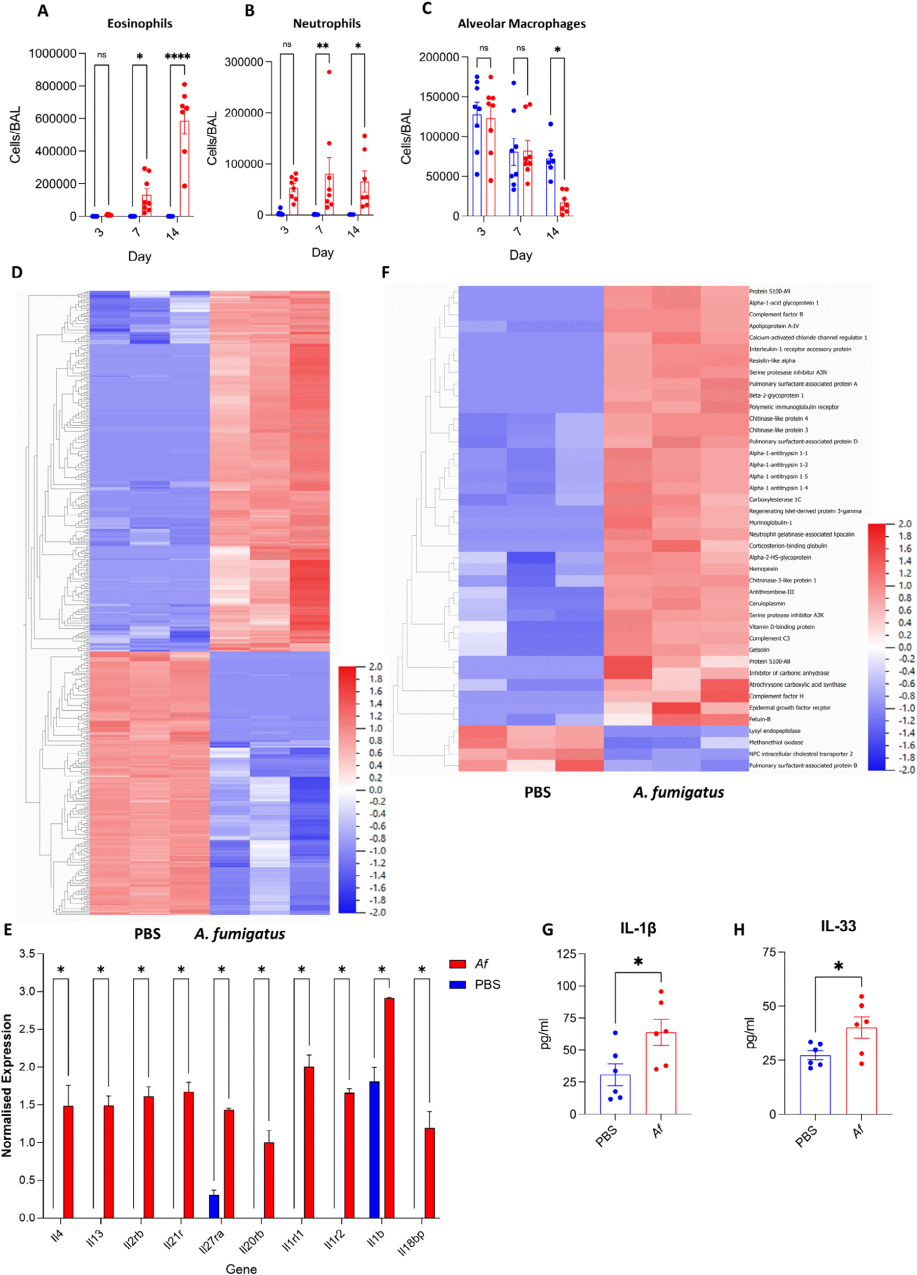
Allergic fungal airways disease is characterised by increased IL‐1 family signalling. Mice were dosed daily with *A. fumigatus* for up to 14 days. Populations of (A) Eosinophils, (B) Neutrophils, (C) Alveolar Macrophages (*n* = 6–8) in BAL were enumerated. (D) RNAseq was carried out on BAL cells (*n* = 3), (D) differential expression heatmap and (E) upregulated interleukins and interleukin receptors. LC–MS/MS analysis was carried out on BAL fluid (*n* = 3) (F) differential expression heatmap and BAL assessed for (F) IL‐1β and (G) IL‐33 levels (*n* = 6). Data represents mean ± SEM from at least two independent experiments. *p < 0.05, **p < 0.01, ****p < 0.0001.

Transcriptomic profiling of bronchoalveolar lavage (BAL) cells after 14 days of exposure identified 443 differentially expressed genes, including upregulation of IL‐1 family receptors (IL‐1r1, IL‐1r2, IL‐1rl1/ST2), IL‐1β, and IL‐18 binding protein, alongside type 2 cytokines such as IL‐4 and IL‐13 (Figure 1D,E). Concurrent increases in lactate dehydrogenase (LDH) and total free protein levels in BAL fluid indicated cell damage and death in the airways (Figure S1J,K). Complementary proteomic analysis revealed elevated DAMPs including calprotectin (S100A8/9), the IL‐1RAP co‐receptor, chitinases (CHI3L1, AMCase), and pulmonary surfactants (Figure 1F). Both IL‐1β and IL‐33 were markedly elevated in BAL fluid (Figure 1G,H), supporting activation of IL‐1 family cytokine pathways.

Functional validation using IL‐1RAP‐deficient mice (IL‐1RAP^−/−^) confirmed its important role in maximal eosinophilic inflammation. IL‐1RAP^−/−^ mice did not show increased severity of infection compared to wild‐type controls (Figure 2A), however, a marked reduction in airway eosinophilia was observed. There was similar neutrophil influx and alveolar macrophage loss in IL‐1RAP^−/−^ mice compared to wild‐type controls (Figure 2B–D), and unchanged fungal burdens (Figure 2E). BAL levels of IL‐33 were unaffected; however, the type 2 effector cytokines IL‐5 and IL‐13 were significantly decreased (Figure 2F–H). Analysis of innate lymphoid cells demonstrated that IL‐1RAP^−/−^ mice failed to expand ILC2 populations following IL‐33 (Figure S2C) or Af lysate challenge (Figure 2I), implicating the IL‐33/IL‐1RL1/IL‐1RAP axis as a major contributor to eosinophilia through ILC2‐derived IL‐5 and IL‐13 which could be therapeutically targeted to reduce allergic inflammation. Of note, recombinant IL‐33 induced a much greater number of ILC2s compared to fungal lysate in wild‐type animals; this is likely due to the direct interaction of IL‐33 with the IL‐1RL1/IL‐1RAP complex, compared to indirect activation by fungal lysate through epithelial damage‐dependent alarmin release.

**FIGURE 2:**
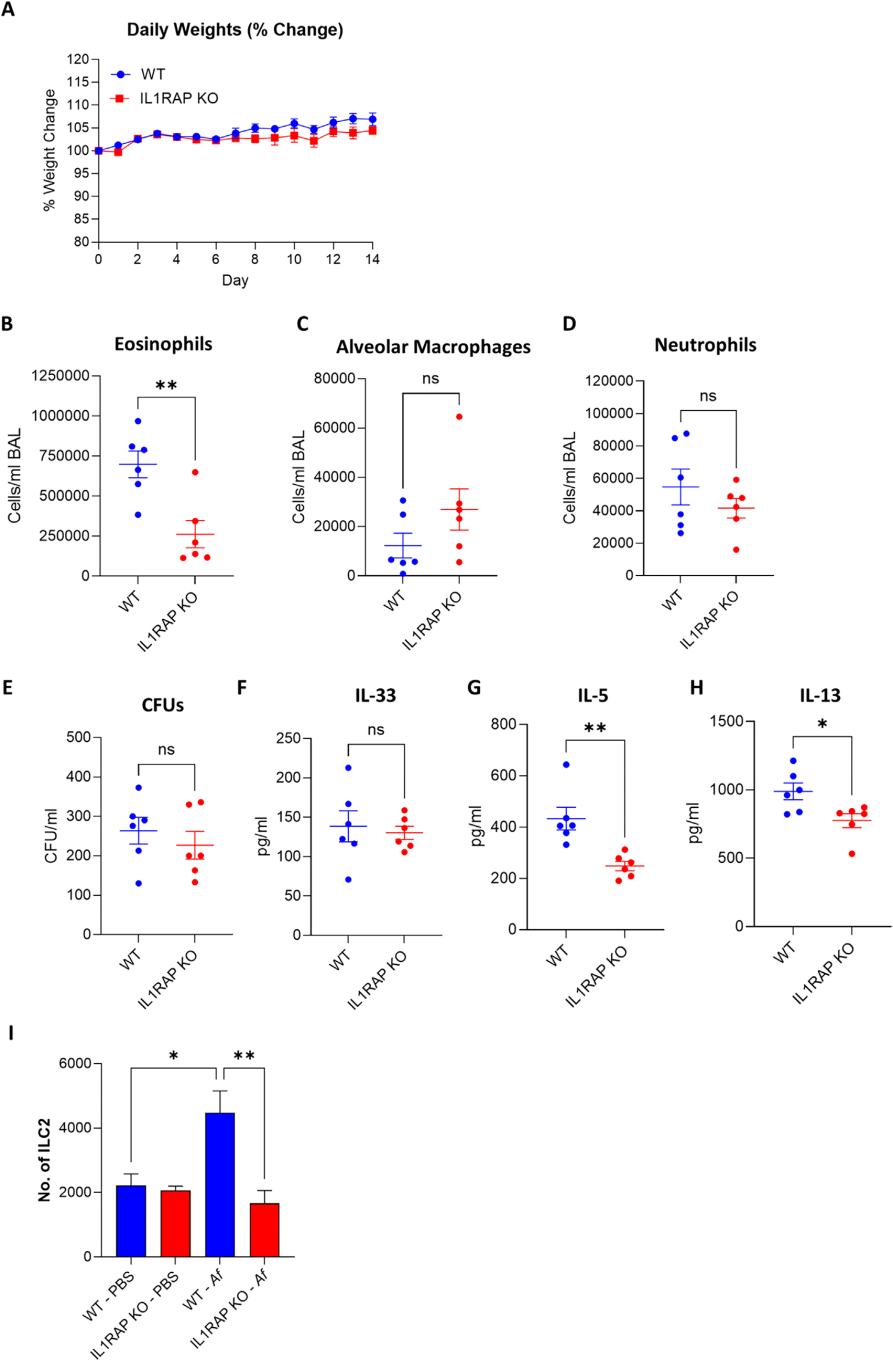
IL‐1RAP deficiency reduces eosinophilia during chronic exposure to *A. fumigatus* . IL1RAP^−/−^ and WT mice were dosed daily with *A. fumigatus* for 14 days. Mice were monitored for (A) weight change. (B–H) Bronchoalveolar lavage fluid (BALF) was assessed for (B) eosinophil, (C) alveolar macrophage and (D) neutrophil counts, (E) CFUs and levels of (F) IL‐33, (G) IL‐5 and (H) IL‐13 (*n* = 6). (I) IL1RAP−/− and WT mice were dosed with *Af* lysate (*n* = 3). Lung homogenates were assessed for ILC2 enumeration. Data represents mean ± SEM from at least two independent experiments. *p < 0.05, **p < 0.01.

In our model of fungal allergic airway disease, IL‐1RAP deficiency resulted in markedly reduced eosinophilia and lower production of the Th2‐associated cytokines IL‐5 and IL‐13. These findings are similar to observations in IL‐1RL1 (ST2) knockout models, where IL‐33‐driven eosinophilic responses were also diminished [4]. IL‐1RAP acts as an essential co‐receptor facilitating IL‐33/IL‐1RL1‐mediated activation of group 2 innate lymphoid cells (ILC2s) and subsequent Th2 cytokine production, processes central to the development of allergic fungal airway disease and ABPA.

From a therapeutic perspective, monoclonal antibodies targeting IL‐1RAP, designed to inhibit signalling across multiple IL‐1 family members, have successfully reduced inflammation in murine models of sterile and cytokine‐driven inflammation [5]. These agents are currently progressing through clinical evaluation [6]. Collectively, these findings highlight IL‐1RAP as an important regulator of type 2 airway inflammation and a promising target for selective immunomodulation in eosinophil‐dominated respiratory diseases such as ABPA.

## Author Contributions

Data collection: T.J.W., J.S.G., L.E.G.‐H., D.B. Data analysis: T.J.W., J.S.G., L.E.G.‐H., D.B., A.K.R., A.S., J.R.N., D.A.‐J. Conceptualization: T.J.W., A.S., J.R.N., D.A.‐J. Writing: T.J.W., J.S.G., L.E.G.‐H., D.B., A.K.R., A.S., J.R.N., D.A.‐J. Supervision: A.K.R., A.S., J.R.N., D.A.‐J.

## Funding

This work was supported by Cystic Fibrosis Trust. Wellcome Trust. National Institutes of Health.

## Conflicts of Interest

The authors declare no conflicts of interest.

## Supporting information


**Figure S1:** Increased inflammation, mucus production and fungal persistence in the lung of mice with allergic fungal airway disease. Mice were dosed daily with 2 × 10^5^ live *A. fumigatus* conidia or PBS for 14 days and culled 24 h after the final dose. Mice were monitored for survival and (A) weight change (*n* = 10). (B–E) Representative lung sections stained for (B) H&E and (D) PAS. (C) %Inflammation and (E) % Mucus was determined by threshold image analysis (*n* = 3). Bronchoalveolar lavage fluid was collected, (F) CFUs were counted (*n* = 4) and flow cytometry was carried out to determine the (G) monocyte, (H) T cell and (I) B Cell numbers in the airways (*n* = 6–8). Bronchoalveolar lavage fluid was assessed for (J) LDH (*n* = 8) and (K) Total protein (*n* = 4). Data represents mean ± SEM from at least two independent experiments. (A) Two‐way ANOVA, (G–I) One‐way ANOVA, (C, E, F, J, K) Students *t*‐test, *p < 0.05, **p < 0.01, ***p < 0.001, ****p < 0.0001. CFU, colony forming unit; H&E, Haematoxylin & eosin; PAS, Periodic acid Schiff.
**Figure S2:** Representative Gating Strategy for immune profiling of murine BAL. Cells were first gated on forward and side scatter followed by the exclusion of doublets. Dead cells were removed from analysis according to a LIVE/Dead stain and immune cells were identified as CD45^+^, cell types were subsequently determined through positive and negative getting of cell specific markers. Cell populations were enumerated using flow cytometry counting beads, identified by forward and side scattered, followed by autofluorescence. Cells were gated for either (A) General immune cell sets or (B) ILC2s, identified as LIN^−^CD90.2^+^CD127^+^GATA3^+^. (C) Bar plot representing the number of ILC2s. One‐way ANOVA, **p < 0.01.

## Data Availability

The data that support the findings of this study are openly available in Sequence Read Archive (SRA) at https://www.ncbi.nlm.nih.gov/sra, reference number PRJNA1250534.
