# Supplementary material for: Selective Targeting of IL‐1RAP‐Dependent Eosinophilic Inflammation in Allergic Fungal Airway Disease
Source: Allergy. 2026 Feb 11;81(4):1302–5. doi: 10.1111/all.70256 (PMC13040660; doi:10.1111/all.70256)
Supplement: Supplementary file 1 — Figure S1: Increased inflammation, mucus production and fungal persistence in the lung of mice with allergic fungal airway disease. Mice were dosed daily with 2 × 10^5^ live A. fumigatus conidia or PBS for 14 days and culled 24 h after the final dose. Mice were monitored for survival and (A) weight change (n = 10). (B–E) Representative lung sections stained for (B) H&E and (D) PAS. (C) %Inflammation and (E) % Mucus was determined by threshold image analysis (n = 3). Bronchoalveolar lavage fluid was collected, (F) CFUs were counted (n = 4) and flow cytometry was carried out to determine the (G) monocyte, (H) T cell and (I) B Cell numbers in the airways (n = 6–8). Bronchoalveolar lavage fluid was assessed for (J) LDH (n = 8) and (K) Total protein (n = 4). Data represents mean ± SEM from at least two independent experiments. (A) Two‐way ANOVA, (G–I) One‐way ANOVA, (C, E, F, J, K) Students t‐test, *p < 0.05, **p < 0.01, ***p < 0.001, ****p < 0.0001. CFU, colony forming unit; H&E, Haematoxylin & eosin; PAS, Periodic acid Schiff. Figure S2: Representative Gating Strategy for immune profiling of murine BAL. Cells were first gated on forward and side scatter followed by the exclusion of doublets. Dead cells were removed from analysis according to a LIVE/Dead stain and immune cells were identified as CD45+, cell types were subsequently determined through positive and negative getting of cell specific markers. Cell populations were enumerated using flow cytometry counting beads, identified by forward and side scattered, followed by autofluorescence. Cells were gated for either (A) General immune cell sets or (B) ILC2s, identified as LIN−CD90.2+CD127+GATA3+. (C) Bar plot representing the number of ILC2s. One‐way ANOVA, **p < 0.01. [file ALL-81-1302-s001.docx]

Supplementary Materials

Methods

*Mouse Ethics and Housing*

All mouse experiments were approved by the United Kingdom Home Office and the Imperial College animal ethics committee, performed in accordance with the project licenses PPL PF9534064 and PP6711863. Male C57BL/6J mice (6-8 weeks old) were ordered from Charles River, UK. B6;129S1-Il1raptm1Roml/J (IL-1RAP^-/-^) were purchased from Jax (RRID:IMSR_JAX:003284) and backcrossed for 10 generations to C57BL/6J. All mice were housed in groups of 3-5 in individually ventilated cages with autoclaved bedding, nesting material, and free access to autoclaved food/water under a 12 h light/dark cycle at 21±2 °C and 55±10% humidity and allowed to acclimate for at least 7 days prior to experimentation. The study used a parallel-group design with 2 (vehicle control, treatment) or 4 groups (WT-vehicle, WT-treatment, KO-vehicle, KO-treatment), the experimental unit was the individual animal. In total, 90 animals were used. Mice were randomly assigned to groups on the day of allocation, with groups mixed among cages where possible. Planned sample sizes were based on prior *Aspergillus* challenge studies where n=6-8 reliably detected relevant effects on lung inflammation and cytokines, exact n numbers are given in the figure legends. Only mice with failed bronchoalveolar lavage (n = 3) were excluded from analysis per predefined criteria.

*Aspergillus culture*

*Aspergillus fumigatus* strain CEA10 (FGSC A1163) was obtained from the Fungal Genetics Stock Centre) and cultured on Sabouraud dextrose agar (Oxoid). Conidia were harvested in 0.1% Tween/H2O and filtered through MIRACLOTH (Calbiochem, UK), washed and stored in PBS.

*Repeat Challenge Aspergillus Model*

Using the approach employed by Porter *et al*^1^, mice were intranasally dosed with PBS or 2x10^5^ resting conidia in 50μl of PBS while under isoflurane anaesthesia. Doses were repeated for 3, 7 and 14 days with mice being culled either 3 or 24 hours after the final dose by intra-peritoneal overdose of pentobarbital.

*ILC2 Expansion*

Using the approach employed by Puttur *et al.*^2^, mice were intranasally dosed with PBS, 10μg (in 25 μl PBS) *Aspergillus fumigatus* lysate or 1μg (in 25 μl PBS) carrier-free recombinant murine rIL-33 (eBioscience) 3 times over the course of 1 week. Mice were culled 24hrs after the final lysate or cytokine dose.

*Histology*

Lungs were inflated, excised and fixed in 10% neutral buffered formalin overnight and moved into 70% ethanol. Lungs were then wax embedded, sectioned to 4μm sections and H&E or PAS stained. %Inflammation and %Mucus were determined by threshold image analysis, using Image J software.

*Bronchoalveolar Lavage*

Bronchoalveolar lavage (BAL) was performed by tracheal cut-down and intubation with a self-designed catheter. Lungs were lavaged by 3 separate instillations of 1ml of PBS/2mM EDTA. The supernatant fraction of the first instillation was used for LDH, Bradford, ELISA and Proteomics. The cell fractions of all 3 instillations were combined and used for flow cytometry. For CFUs all instillations were combined.

*Colony Forming Unit Assay*

Serial dilution of BAL fluid was performed and plated onto Sabouraud dextrose agar plates in triplicate. Plates were incubated for 18-24 hrs at 37°C and CFUs were manually counted.

*Lung Homogenate Preparation*

For homogenisation lungs were excised, minced and incubated with RPMI containing 0.25 mg/mL Liberase™ TM Research Grade (Merck, Catalog #5401119001) and DNase I (Merck, Catalog #10104159001) in a shaking incubator at 37°C for 30 minutes. Digested lung was then passed through a 70μm cell strainer and centrifuged to form a cell pellet.

*Flow Cytometry*

Cell pellets were stained for either immune cells with: Live/Dead Blue (L34959, Thermo Fisher Scientific), CD19-BV785 (115543, Biolegend), CD45.2-BUV737 (564880, BD), CD3-BUV395 (740268, BD), Ly6C-PE-Cy7 (25-5932-82, eBioscience), MHC-II-I/A I/E-APC-Cy7 (107628, Biolegend), Ly6G-AF700 (127622, Biolegend), CD11b-BV605 (101237, Biolegend), F4/80-APC (17-4801-82, Invitrogen), CD11c-FITC (557400, BD), Siglec F-PE (562068, BD), or for ILC2s with Live/Dead Blue (L34959, Thermo Fisher Scientific), CD45.2-BUV737 (564880, BD), LIN-BV421 (133311, Biolegend), CD90.2-BV605 (140317, Biolegend), CD127-PE-Cy7 (135014, Biolegend), GATA3-AF488 (560163, BD). Cells were fixed in 2% formaldehyde (28908, Thermo Fisher Scientific) and resuspended in FACS buffer (PBS, 1% FBS, 3mM EDTA). 123count eBeads counting beads (01-1234-42, Thermo Fisher Scientific) were used for cell enumeration. Samples were gated according to Fig S2. Flow Cytometry was performed on an LSR Fortessa III flow cytometer (BD Biosciences). Data were analysed using FlowJo (Treestar).

*Bradford Assay*

Basic protein quantification of BAL supernatant was carried out using Pierce Coomassie Plus (Bradford) Assay Reagent (23238, ThermoFisher Scientific), concentrations were calculated based on a BSA standard curve.

*Transcriptomics*

Bulk RNA was isolated from total bronchoalveolar lavage cells. Library preparation and sequencing was carried out by Novogene (Novogene (UK) Ltd). Reads were mapped to the murine genome (GRCm39/mm39, GCF_000001635.27). Analysis of RNAseq libraries was carried out using the Qlucore Multiomics Explorer 3.9 (Qlucore, Sweden). Genes with an adjusted P-value <=0.05 and fold change >2 were assigned as differentially expressed. The RNA-seq data generated during this study are available at NCBI Sequence Read Archive: PRJNA1250534.

*Proteomics*

BAL supernatant was processed using a Multiple Affinity Removal Spin Cartridge Mouse 3 (5188-5289, Agilent) according to the manufacturer's instruction. Samples then underwent acetone precipitation and trypsin/lys C digest and were stored at -80°C until analysis.

All samples were injected and separated on a 1290 liquid chromatography system (Agilent) and extracted into peak lists using SpectrumMill (Agilent). Analysis of proteomic results was carried out using Qlucore Multiomics Explorer 3.9 (Qlucore, Sweden).

*Lactate Dehydrogenase (LDH) Assay*

LDH levels in BAL supernatants were measured using the CytoTox 96® Non-Radioactive Cytotoxicity Assay kit (Promega) according to the manufacturer’s instructions.

*ELISA*

ELISAs were performed according to the manufacturer’s instruction using the murine IL-1β, IL-33, IL-5 and IL-13 DuoSet kits from R&D systems.

*Statistics*

All data are expressed as mean ± SEM unless otherwise stated. To assess statistical significance, one-way ANOVA (≥3 groups), two-way ANOVA and Students t-test/Mann-Whitney test (2 groups) were carried out. For all figures, p values are represented as followed: *P<0.05, **P<0.01, ***P<0.001, ****P<0.0001. Statistical analyses were carried out using GraphPad Prism (La Jolla, CA, USA) or Qlucore Multiomics Explorer 3.9 (Qlucore, Sweden).

References

(1) Porter PC, Roberts L, Fields A, Knight M, Qian Y, Delclos GL, et al. Necessary and sufficient role for T helper cells to prevent fungal dissemination in allergic lung disease. *Infection and immunity.* 2011; 79 (11): 4459–4471. 10.1128/IAI.05209-11.

(2) Puttur F, Denney L, Gregory LG, Vuononvirta J, Oliver R, Entwistle LJ, et al. Pulmonary environmental cues drive group 2 innate lymphoid cell dynamics in mice and humans. *Science immunology.* 2019; 4 (36): eaav7638. doi: 10.1126/sciimmunol.aav7638. 10.1126/sciimmunol.aav7638.

Supplementary Figures


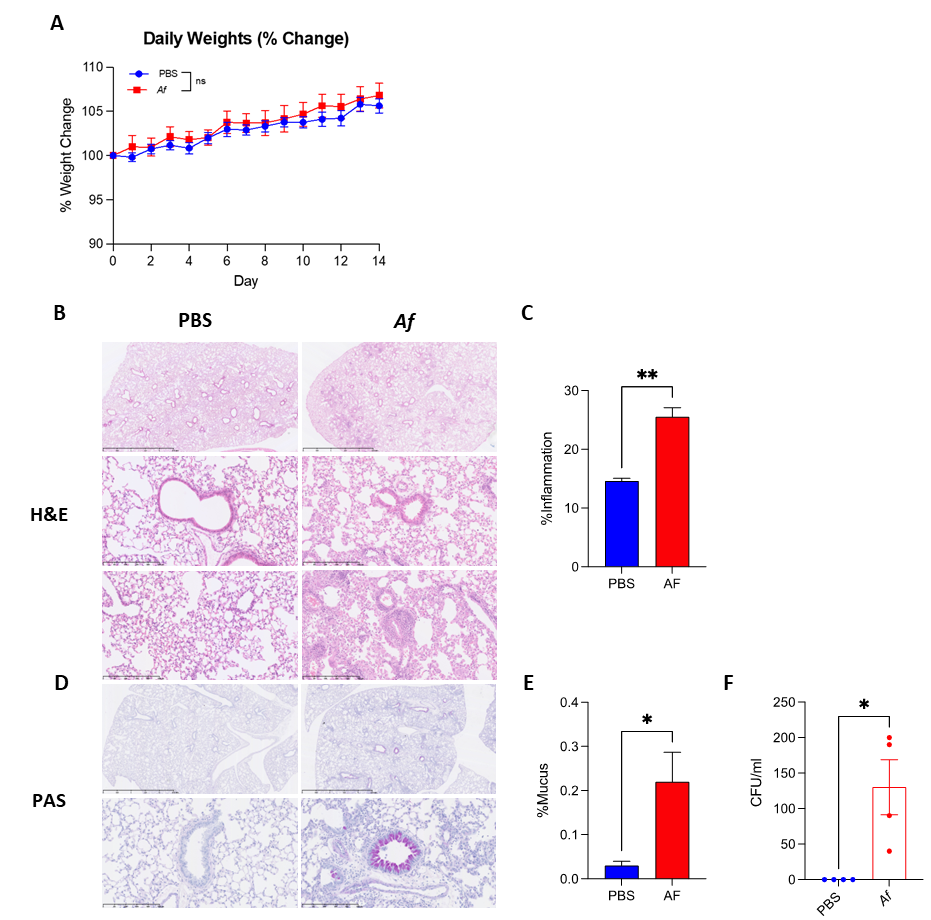

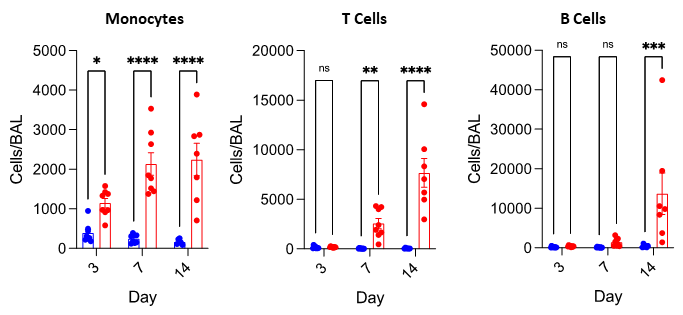


**G**

**H**

**I**

**J**

**K**

**Figure S1: Increased inflammation, mucus production and fungal persistence in the lung of mice with allergic fungal airway disease.** Mice were dosed daily with 2x10^5^ live *A. fumigatus* conidia or PBS for 14 days and culled 24 hours after the final dose. Mice were monitored for survival and **A)** weight change (n=10). **B-E)** Representative lung sections stained for **B)** H&E and **D)** PAS. **C)** %Inflammation and **E)** % Mucus was determined by threshold image analysis (n=3). Bronchoalveolar lavage fluid was collected, **F)** CFUs were counted (n=4) and flow cytometry was carried out to determine the **G)** monocyte, **H)** T cell and **I)** B Cell numbers in the airways (n=6-8). Bronchoalveolar lavage fluid was assessed for **J)** LDH (n=8) and **K)** Total protein (n=4). Data represents mean ± SEM from at least two independent experiments. A) Two-way ANOVA, G-I) One-way ANOVA, C, E, F, J, K) Students T-test, *P<0.05, **p<0.01, ***P<0.001, ****P<0.0001. CFU – Colony forming unit, H&E – Hematoxylin & eosin, PAS – Periodic acid Schiff.


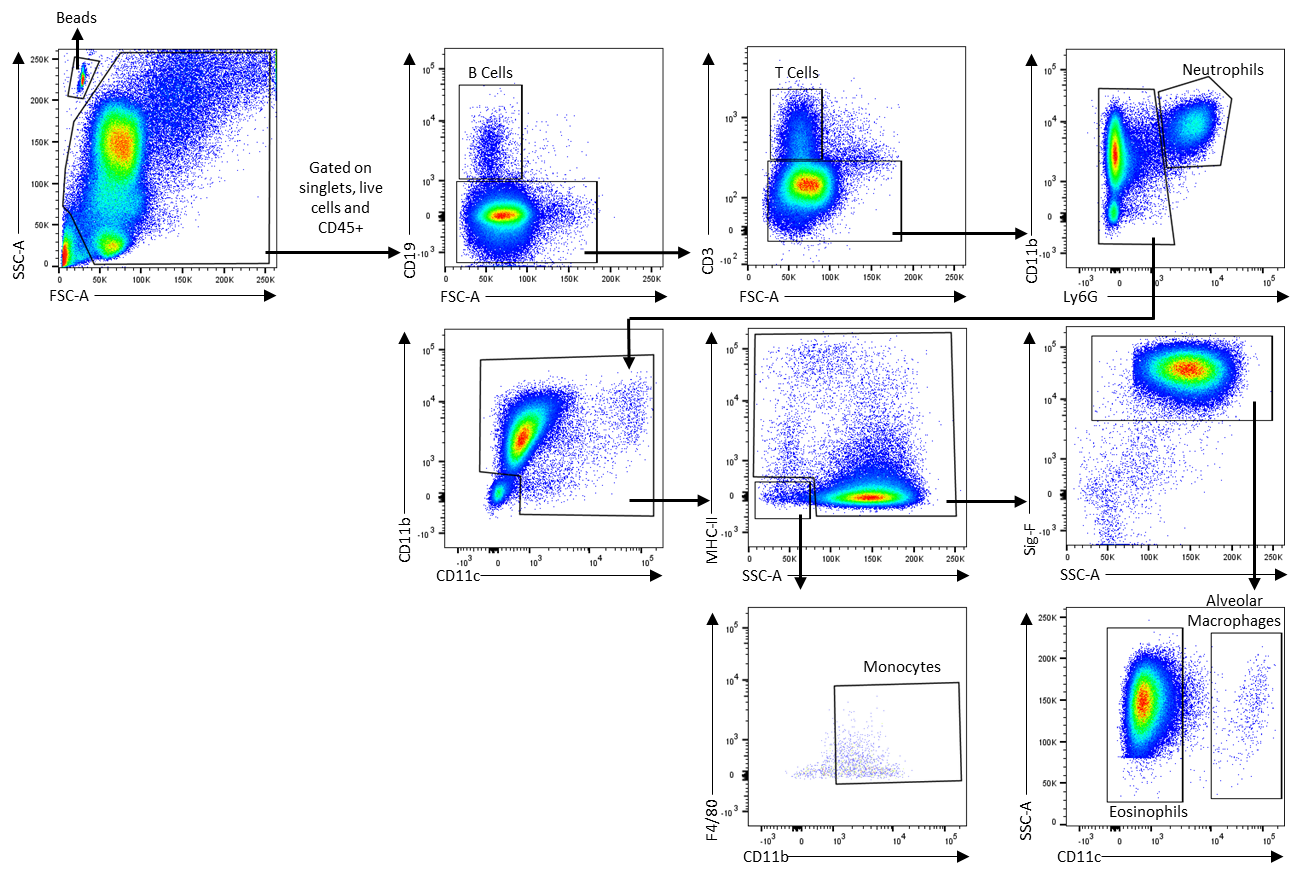

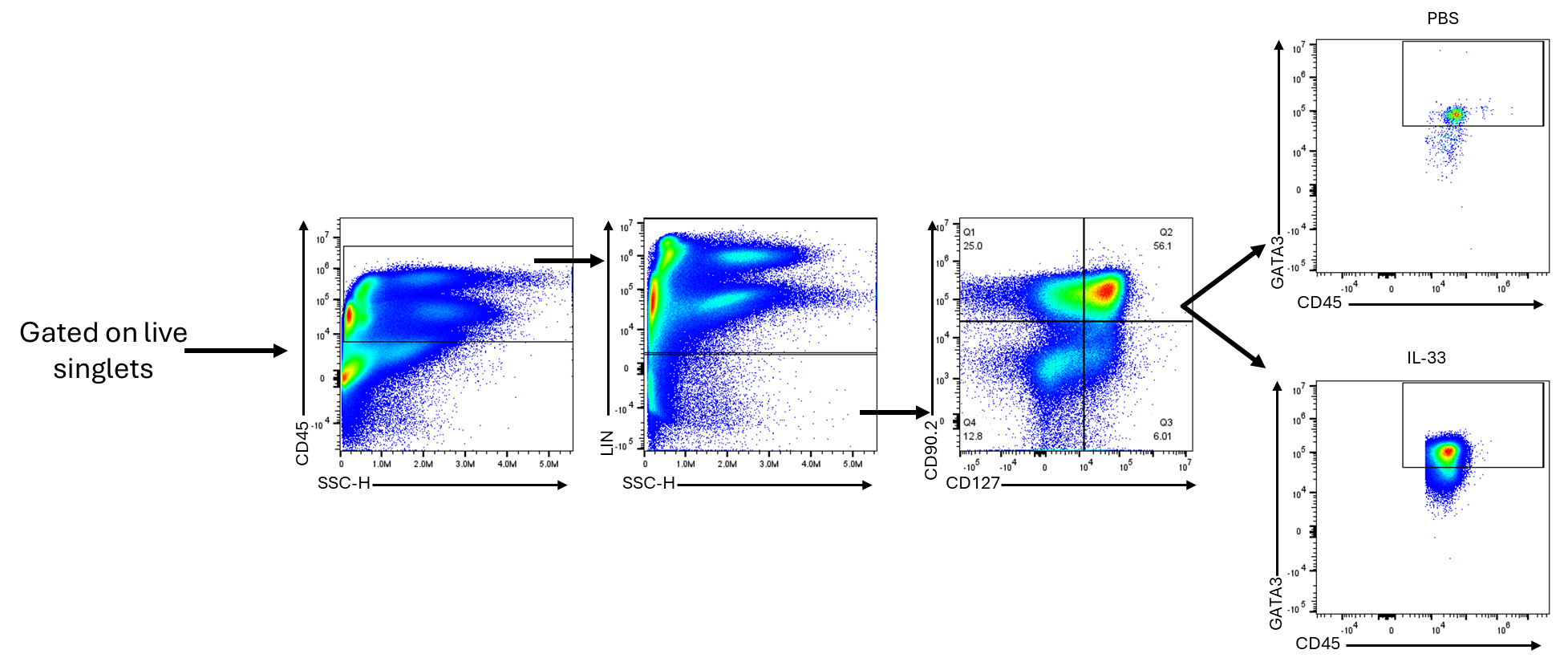


**A**

**B**

**C**

**Figure S2: Representative Gating Strategy for immune profiling of murine BAL.** Cells were first gated on forward and side scatter followed by the exclusion of doublets. Dead cells were removed from analysis according to a LIVE/Dead stain and immune cells were identified as CD45+, cell types were subsequently determined through positive and negative getting of cell specific markers. Cell populations were enumerated using flow cytometry counting beads, identified by forward and side scattered, followed by autofluorescence. Cells were gated for either **A)** General immune cell sets or **B)** ILC2s, identified as LIN^-^CD90.2^+^CD127^+^GATA3^+^. **C)** Bar plot representing the number of ILC2s. One-way ANOVA, **P<0.01.
